# Association of the Matrix Attachment Region Recognition Signature with coding regions in *Caenorhabditis elegans*

**DOI:** 10.1186/1471-2164-8-418

**Published:** 2007-11-15

**Authors:** Alasdair Anthony, Mark Blaxter

**Affiliations:** 1 Institute of Evolutionary Biology, School of Biological Sciences, University of Edinburgh, Edinburgh EH9 3JT, UK

## Abstract

**Background:**

Matrix attachment regions (MAR) are the sites on genomic DNA that interact with the nuclear matrix. There is increasing evidence for the involvement of MAR in regulation of gene expression. The unsuitability of experimental detection of MAR for genome-wide analyses has led to the development of computational methods of detecting MAR. The MAR recognition signature (MRS) has been reported to be associated with a significant fraction of MAR in *C. elegans *and has also been found in MAR from a wide range of other eukaryotes. However the effectiveness of the MRS in specifically and sensitively identifying MAR remains unresolved.

**Results:**

Using custom software, we have mapped the occurrence of MRS across the entire *C. elegans *genome. We find that MRS have a distinctive chromosomal distribution, in which they appear more frequently in the gene-rich chromosome centres than in arms. Comparison to distributions of MRS estimated from chromosomal sequences randomised using mono-, di- tri- and tetra-nucleotide frequency patterns showed that, while MRS are less common in real sequence than would be expected from nucleotide content alone, they are more frequent than would be predicted from short-range nucleotide structure. In comparison to the rest of the genome, MRS frequency was elevated in 5' and 3' UTRs, and striking peaks of average MRS frequency flanked *C. elegans *coding sequence (CDS). Genes associated with MRS were significantly enriched for receptor activity annotations, but not for expression level or other features.

**Conclusion:**

Through a genome-wide analysis of the distribution of MRS in *C. elegans *we have shown that they have a distinctive distribution, particularly in relation to genes. Due to their association with untranslated regions, it is possible that MRS could have a post-transcriptional role in the control of gene expression. A role for MRS in nuclear scaffold attachment is not supported by these analyses.

## Background

As genome sequencing and annotation has progressed, it has become clear that even relatively compact eukaryotic genomes have large amounts of non-coding DNA. This DNA harbours elements that control genomic activity such as gene regulators, non-coding RNAs and less well characterised elements that position the chromosomes on the nuclear matrix. The nuclear matrix forms a three dimensional protein network onto which chromatin fibres are attached. Interaction between chromatin and the nuclear matrix is believed to occur at specific sites from 300 bp to several kb long, termed matrix attachment regions (MAR) [1].

There is increasing evidence for the involvement of MAR in gene regulation. For example, expression levels of some genes alter depending on their position relative to the matrix [2]. MAR have also been associated with enhanced transcription, notably in transgene constructs where flanking transgenes with MAR results in higher and more stable expression (for review see [3]). A role for MAR as a boundary between functional chromatin domains has been proposed [4,5]. The effects of long-range enhancers may be restricted by the positioning of MAR [6]. MAR have also been implicated in the positioning of chromosomal territories [4,7]. Coordinated spatial positioning of sequences on different chromosomes can facilitate interactions *in trans*. For example, active genes from different chromosomes have been shown to migrate through the nuclear space to converge on "transcriptional factories" [8]. Localisation of genes in this way is likely to involve control of higher order chromosome structure and there is evidence that some chromatin loop attachments are under developmental control [9].

Experimentally, MAR have been defined as either DNA fragments that remain bound to the nuclear matrix after chromatin proteins and other DNA have been removed, or DNA that binds to extracted nuclear matrix in the presence of competitor DNA [10,11]. The most common experimental method for identifying MAR uses re-association assays to define DNA fragments that bind to the nuclear matrix [12]. However, as experimental methods are poorly amenable to genome wide analysis, computational methods have been sought for identifying MAR.

MAR-associated sequences for approximately 500 experimentally defined MAR are catalogued in the S/MAR transaction Database [13]. The overriding feature of many MAR is that they are AT rich, but several other more specific sequence motifs have also been identified [4]. MAR sequences also show elevated DNA unwinding potential, through stress-induced DNA duplex destabilisation (SIDD) [14]. Computational tools based on these sequence characteristics have been used to identify MAR using DNA sequence information alone. MARfinder uses 20 motifs within a set of higher order rules. The density of rule occurrences is then used to identify MAR [15,16]. SMARTest is based on a density analysis of a set of MAR sequences represented by position weight matrices [17]. An *in silico*, genome-wide mapping of MAR in *Arabidopsis thaliana *using SMARTest revealed that genes containing predicted MARs had low transcription levels [18]. SIDD identifies putative MAR based on the predicted sites of stress-induced DNA duplex destabilisation [14]. ChrClass uses multivariate linear discriminant analysis to compare MAR sequences and develop a classification system [19]. The limited effectiveness of these methods in reliably identifying MAR is discussed in a recent comparative study of MAR prediction software [20].

The most complex motif associated with MAR sequences is the bipartite MAR recognition signature (MRS). The MRS was identified through analysis of MAR from three independent genomic regions of >30 kb in *A. thaliana *[5]. To assess the effectiveness of the MRS, van Drunen *et al*. mapped all the MRS and experimentally detected MAR on a single *C. elegans *genomic DNA segment, ~40 kb long. All MRS were located in six of the seven MAR sites. Further analysis of >300 kb of genomic sequence from 7 other eukaryotic organisms showed that MRS were present in 80% of MAR, leading van Drunen *et al*. to suggest that the MRS was a specific sequence element representative of a subset of MAR [5].

Donev *et al*. used the MRS to identify novel MAR in the human major histocompatibility complex class II region [21]. The regions they identified were found to bind the nuclear matrix and a subset were also able to bind the mRNA processing protein hnRNP-A1 during transcriptional up-regulation of nearby genes. The MRS has also been used to identify MAR in *Entamoeba histolytica *and was found in MAR from *Bombyx mori *[22,23]. However, MAR mapping studies in mammals have shown that MRS are sometimes identified outside known MAR sites [24]. In their analysis of 1 Mb of the mouse genome, Purbowasito *et al*. reported that MAR prediction based on MRS had a specificity of 41%, with 29 of 49 predictions lying outside experimentally defined MAR [25]. There is, therefore, some doubt as to the effectiveness of the MRS as a marker for MAR.

We have undertaken a genome-wide mapping and analysis of MRS in *C. elegans *in an attempt to determine the validity of the MRS. If MRS constitute a feature with real biological meaning their distribution would be expected to be non-random with respect to other genome features. We found that the MRS signature had a distinctive pattern of distribution along chromosomes, similar to that of genes. Further, we show that there is a marked increase in the frequency of MRS in the regions flanking *C. elegans *coding sequence (CDS).

## Results

The MRS is a degenerate bipartite motif consisting of a 16 bp pattern, AWWRTAANNWWGNNNC (where W = A or T, R = A or G, N = A,C,G or T), within which one mismatch is allowed, and an 8 bp pattern, AATAAYAA (where Y = C or T) [5]. To be scored as an MRS, both these sequences must lie within 200 bp of each other, although they may overlap and they may be on either strand of the DNA duplex [5]. Existing MRS finding programs were designed to under-report closely apposed MRS [26]. To allow full control over data reported, a custom program, MRSfinder, was designed. MRSfinder was used to map the location of MRS across the entire *C.elegans *genome.

MRS were found across all 6 *C.elegans *chromosomes at an average frequency of 249 per Mb, similar to the frequency of genes (228 per Mb). At small scales (<100 kb), the motif distribution was noisy (see Additional File 1). As would be expected of an AT-rich motif, there was some correlation with regions of high AT% (see below).

**Figure 1:**
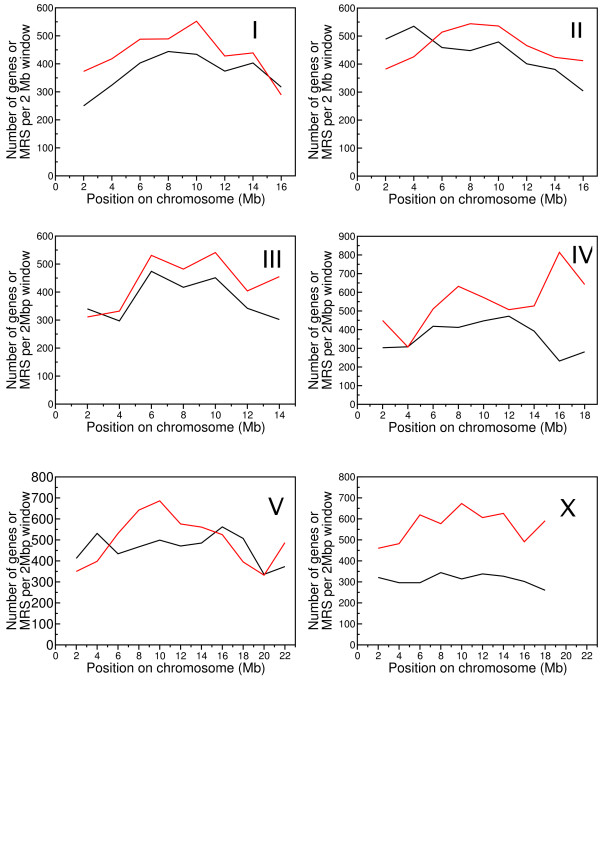
**Distribution of genes and MRS in *C. elegans *chromosomes**. Number of gene (black) and MRS (red) start positions in non-overlapping 2 Mb windows. To account for short sequence length in the end window, the number of genes and MRS in the last window has been scaled to 2 Mb.

However, at a chromosomal level distinct patterns emerged. Analyses of non-overlapping 2 Mb windows along the chromosomes showed that MRS were significantly more abundant in the centres than in the arms of all chromosomes except chromosome IV (Figure 1 and Additional File 2). The division between chromosome arms and centres is characteristic of several genomic features in *C. elegans*. Centres tend to be gene rich, with a high concentration of essential, well conserved and highly expressed genes [27,28]. By comparison, the chromosome arms exhibit a higher meiotic recombination rate, and are enriched for transposons and repeats [27]. Thus, at the chromosome level, MRS are more likely to be found in the vicinity of highly expressed and essential genes.

### MRS frequency in real sequence is different to that in randomised sequence

**Figure 2:**
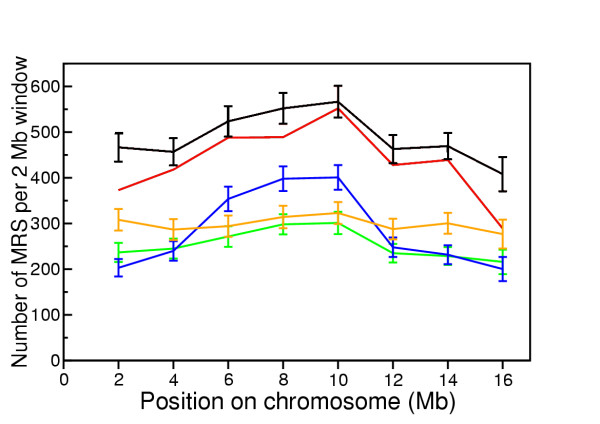
**Comparison of MRS distribution in *C. elegans *chromosome I under various randomisations**. The number of MRS in non-overlapping 2 Mb windows in real *C. elegans *chromosome I sequence is shown in red. The chromosome was randomised in non-overlapping 2 Mb sections using four different Markov chain processes. The average number of MRS +/- one standard deviation for the 2 Mb windows for zero (mononucleotide, black), first (orange), second (green) and third (blue) order Markov chain process randomisation is shown.

Although the distribution of MRS appeared to correlate broadly with several other genome features, the specific nucleotide composition of each sequence window will influence the number of MRS. By randomising the genome sequence whilst maintaining nucleotide composition (mononucleotide randomisation), we estimated the number of MRS expected in the sequence due to nucleotide composition alone. Additional randomisation models were used in order to account for relationships between adjacent bases. The mononucleotide randomisation model generated sequence in which the frequency of each of the four nucleotides matched that observed in the chromosomal sequence. More complex first, second and third order Markov chain randomisation processes reflected the di-, tri- and tetra-nucleotide content of the chromosomal sequence. For each 2 Mb non-overlapping window used in Figure 1, the nucleotide sequence was randomised 1000 times, and MRSfinder was used to map and count the number of MRS in each randomised sequence. A comparison of MRS counts for chromosome I under each randomisation process is shown in Figure 2 (results for second order Markov chain randomisation of the other chromosomes can be found in Additional file 3). The observed number of MRS in mononucleotide randomised sequence was similar to that found in real sequence, while the first, second and third order Markov chain randomised sequence yielded far fewer MRS. As MRS occurrence was best modelled by the mononucleotide randomisation process, subsequent analyses focussed on this method of randomisation.

**Figure 3:**
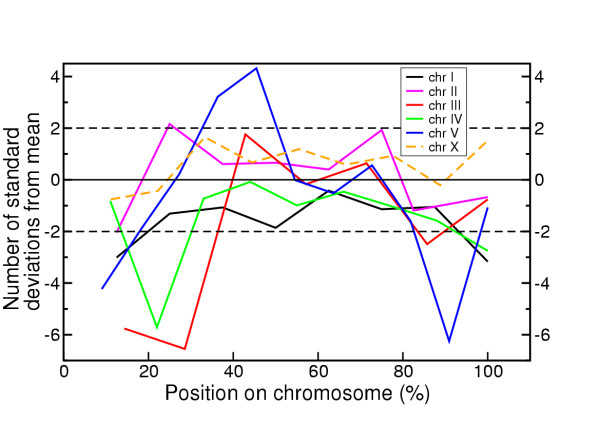
**Distribution of MRS along *C. elegans *chromosomes, relative to average number of MRS in chromosome sequence randomised in 2 Mb sections**. The sequence of each chromosome was randomised using a mononucleotide process in non-overlapping sections of 2 Mb, MRS were then mapped in this sequence using MRSfinder. This was repeated 1000 times and the average and standard deviation of MRS frequency in the 2 Mb sections was obtained. This graph shows the distribution of MRS in actual *C. elegans *sequence, as the number of standard deviations from the mean MRS frequency in the randomised sequence.

Figure 3 shows the difference in observed MRS count for each 2 Mb window from the mean count in the mononucleotide randomised sequences, in terms of standard deviations from the mean. Throughout the length of each chromosome, the number of MRS in real sequence was generally lower than in the mononucleotide randomised sequence. The arms were particularly poor in MRS and the chromosome centres were at most only slightly enriched for MRS. In contrast to the autosomes, the distribution of MRS along chromosome X (Figure 3, broken line) was much more even and similar to that found in mononucleotide randomised chromosome X sequence.

**Figure 4:**
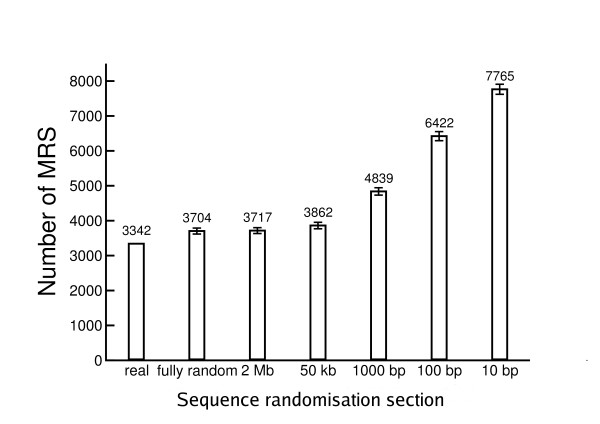
**Frequency of MRS in *C. elegans *chromosome I and various randomisations of it**. Chromosome I was randomised using a mononucleotide process in non-overlapping sections of various lengths 10 bp, 100 bp, 1 kb, 50 kb, 2 Mb and the entire length of the chromosome, and MRSfinder used to identify MRS in each sequence. The randomisation and MRS mapping was repeated 1000 times for each section length. The bar height shows the average number of MRS in the chromosome and the error bars represent +/- 1 standard deviation. The actual number of MRS in *C. elegans *chromosome I is shown for comparison.

One effect of randomising the genome sequence in relatively large sections of 2 Mb is that nucleotide content (or nucleotide local pattern) becomes more uniform across each section, eliminating, for example, local peaks of very high AT%. To identify the effects of local areas of extreme nucleotide composition, mononucleotide randomisation was applied to smaller sections of sequence (10 bp, 100 bp, 1 kb, 50 kb, 2 Mb and the whole chromosome length) to *C. elegans *chromosome I. The number of MRS found in the whole chromosome under each mononucleotide randomisation regime, averaged over 1000 iterations, is shown in Figure 4. The numbers of MRS found when the chromosome was randomised along its entire length in one section and in 50 kb sections were very similar to the 2 Mb randomised sequence (about 10% higher than in the actual sequence). However, at randomisation sections of less than 50 kb the total number of MRS found rose dramatically. A similar effect was observed in the second order Markov chain process randomised sequence (data not shown). Compared to actual genomic sequence, the average number of MRS observed in mononucleotide randomised sequence doubled when the chromosome was randomised in sections of 10 bp.

### Different genome feature types have different MRS frequencies

The above results show that the number and distribution of MRS in the *C. elegans *genome is distinct from that found in random sequence. To investigate how this distribution is related to other genome features, the degree of overlap between MRS and different functional parts of the genome was assessed. The number of MRS occupying the same genome space as exons, introns, 3' untranslated regions (UTR), 5' UTR, genes and intergenic regions, is given in Table 1. The expected score indicates how many MRS would be expected to lie in a feature, based on the total size of the feature and assuming a uniform distribution of MRS across the genome.

**Table 1:** Number of MRS in genic and non-genic portions of the genome.

	genes	exons	introns	5' UTR	3' UTR	intergenic
Size of feature (bp)	58734823	25497325	30586607	456649	1616413	41740777
Number of features in genome	18719	124049	100853	8293	9103	18832
Feature AT%	63	57	68	60	68	66
Actual number of MRS in feature	11368	1955	7094	139	691	12683
Expected number of MRS in feature	14303	4218	5883	33	246	10070
Ratio (actual/expected)	0.79	0.46	1.21	4.22	2.81	1.26
AT% corrected ratio (score system 1)	1.05	1.66	0.74	8.80	1.71	1.02
AT% corrected ratio (score system 2)	1.09	1.83	0.64	2.08	1.34	1.03
AT% corrected ratio (score system 3)	1.07	1.77	0.68	2.98	1.46	1.02
AT correction factor	0.76	0.28	1.64	0.48	1.64	1.24

The number of MRS overlapping genes, exons, introns, 3' UTR, 5' UTR and intergenic regions of the genome was used to calculate an overlap score as described in Methods. The expected overlap score was calculated assuming a uniform distribution of MRS across the genome, using the formulae described in Methods. The ratio of the actual to expected score is shown. This ratio was multiplied by the AT correction factor (see Methods) to give the AT corrected ratio. Score system 1 was used except where indicated otherwise.

The ratios of actual and expected MRS numbers showed large differences in MRS abundance in each of the genome features. MRS were particularly rare in exons, which contained less than half the MRS expected. As a result, the number of MRS in genes was also lower than expected, despite enrichment for MRS in introns and untranslated regions. Intergenic regions had slightly more MRS than expected. However, the 5' UTR and 3' UTR were by far the most MRS-enriched parts of the genome, by factors of 4.2 and 2.8 respectively. The relative enrichment of introns, 5' UTR and 3' UTR for MRS provides an explanation for the spatial relationship between genes and MRS described in Figure 1.

The MRS is AT rich and so is more likely to occur in AT rich sequence (see Additional File 4). To control for this bias, an AT-correction factor was used to adjust the expected number of MRS. The correction factor was based on the number of MRS found in mononucleotide random sequence with AT content equivalent to that of each feature, as a proportion of the number of MRS found in random sequence with AT content equivalent to that of the whole genome. When this correction is applied, the AT-poor exons appeared enriched for MRS, while the AT-rich introns had fewer than expected. Both genes and intergenic regions had approximately the number of MRS expected.

However, even with AT correction, the untranslated regions, particularly the 5' UTR, showed strong enrichment for MRS. Alternative overlap scoring systems that take into account partial MRS-feature overlaps did not affect these results. Although UTR form only a small part of the genome and contain only a small proportion of the total MRS, the degree of MRS enrichment and their proximity to genes points to a functional role for MRS.

### Striking peaks of MRS and AT content at CDS boundaries

**Figure 5:**
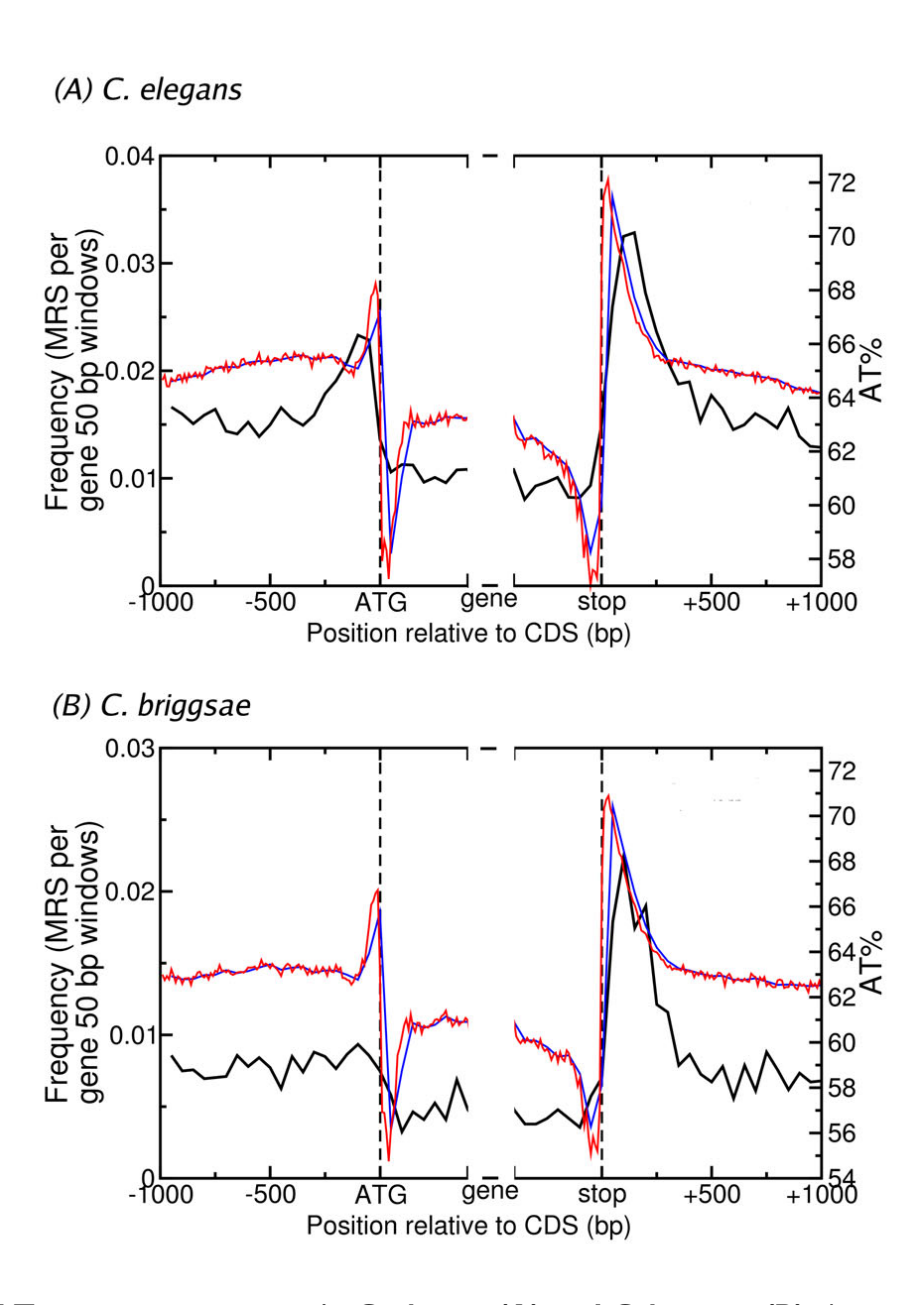
**MRS distribution and AT content near genes in *C. elegans *(A) and *C. briggsae *(B)**. Average AT% in 50 bp (blue line) and 10 bp (red line) non-overlapping windows and number of MRS per CDS in 50 bp non-overlapping windows (black line) is displayed. The windows extend from 1000 bp upstream of the translation start site (ATG codon) through the first 400 bp of the CDS and from the last 400 bp of the CDS, through to 1000 bp downstream of the translation stop site (stop codon).

To clarify the relationship between genes, especially their 5' and 3' UTRs and MRS, the frequency of MRS in the regions surrounding gene boundaries was investigated. Using the data from MRSfinder, MRS locations were plotted on a section of sequence extending 1000 bp upstream of the translation start site (ATG codon) through the first 400 bp of the coding sequence (CDS) from each *C. elegans *gene. The same analysis was carried out on sequence from the last 400 bp of the CDS through to 1000 bp downstream of the stop codon (Figure 5A).

As expected from the overlap of MRS with genes and intergenic regions reported in Table 1, the frequency of MRS in regions outside the CDS was higher than in the CDS itself. The enrichment of MRS in the 5' and 3' UTRs shown in Table 1 correlates with striking increases in MRS frequency in the regions immediately flanking genes. The MRS frequency sharply rose and fell over a span of 350 bp, peaking 50–100 bp upstream of the CDS start. At the 3' end of the CDS the MRS frequency spike had an even greater amplitude, increasing by more than 3 fold in 200 bp.

One explanation for the MRS spikes bounding CDS is that they are related to AT content of these areas. For example, in the case of 3' UTR the apparent over-representation of MRS was reduced when AT content was taken into account (Table 1). Plotting AT content in the region surrounding CDS revealed a pattern of sharp spikes similar to that observed for MRS frequency (Figure 5). However, on closer inspection there were subtle differences between the MRS frequency and AT content variation. Firstly, the upstream AT peak occurred in the 50 bp immediately preceding the start codon, 50–100 bp after the MRS peak. Similarly at the downstream end, the AT peak occurred in the 50 bp immediately following the stop codon, again 50–100 bp separate from the MRS peak.

Another difference was that the AT content dropped to 58% in the first 50 bp of the CDS, then rose to about 62% for the middle part of the CDS. The pattern was similar at the end of the CDS, where the AT dropped to near 58% in the last 50–100 bp. In both locations this AT dip was not matched by a dip in the MRS frequency. The variation in AT content in the vicinity of gene boundaries is an intriguing observation. A similar pattern was described previously by Zhang et al. [29] but further discussion of this phenomenon is beyond the scope of this paper.

An analysis of the MRS frequency surrounding gene boundaries was also performed on a related nematode, *Caenorhabditis briggsae *(Figure 5B). As in *C. elegans*, the frequency of MRS was higher in *C. briggsae *intergenic regions near genes than in CDS. However, from 1 kb upstream to 1 kb downstream of the CDS, the frequency of MRS was generally lower in *C. briggsae *than in *C. elegans*. The main difference in the pattern of MRS frequency between the species was that while *C. briggsae *displayed the same striking increase in average MRS frequency at the 3' end of the CDS, it lacked any increase in frequency at the 5' end. The possibility that less robust gene annotation in *C. briggsae *could have lead to this discrepancy was addressed by filtering the dataset to ensure all CDS started with ATG and ended with a stop codon, and that the selected sequence was complete and of high quality (i.e. no Ns). However, the possibility that the *C. briggsae *gene set is systematically lacking upstream exons cannot be excluded.

The difference between MRS frequency and AT content is even more marked in *C. briggsae *than in *C. elegans*. Although *C. briggsae *lacked an upstream MRS peak, an increase in AT content from about 63% to 66% was evident in the 50 bp immediately preceding the CDS start. In common with *C. elegans*, the downstream AT peak occurred 50 bp before the MRS peak and the AT dip at the start and end of the CDS was not matched by a dip in MRS frequency.

### MRS conservation between *C. elegans *and *C. briggsae*

The distinctive increase in MRS frequency at the downstream end of both *C. elegans *and *C. briggsae *CDS could be due to conservation of MRS in specific genes, or simply a reflection of a general tendency. To investigate this, the occurrence of MRS within 200 bp of the CDS stop codon in *C. elegans *genes was compared to MRS occurrence in the same region of the corresponding *C. briggsae *ortholog (Table 2). Surprisingly, of the 224 *C. briggsae *genes annotated as orthologs of *C. elegans *genes with an MRS within 200 bp of the CDS stop codon, only 18 had an MRS in a similar position. Nonetheless, a small but significant degree of correlation between *C. elegans *genes and their *C. briggsae *orthologs for the presence or absence of MRS was detected (log odds ratio = 0.641, *p *value = 0.006). Therefore, the peak of average MRS frequency at the downstream end of *C. elegans *and *C. briggsae *CDS was due partly to apparent conservation of MRS in specific genes.

**Table 2:** MRS within 200 bp downstream of translation stop sites of *C. briggsae *orthologs of *C.elegans *genes.

		Number of *C. elegans *genes in ortholog set	
		MRS within 200 bp of CDS stop	No MRS within 200 bp of CDS stop

	MRS within 200 bp of CDS stop	18	172
Number of *C. briggsae *genes in ortholog set			
	No MRS within 200 bp of CDS stop	206	3736

The filtered set of *C. elegans *and *C. briggsae *orthologs were assessed to identify the number of genes in the set from each organism that had an MRS within 200 bp of the CDS stop codon. The association between orthologs for the presence or absence of 3' MRS was significant (log odds 0.641, *p *value = 0.006).

### Functional classification of MRS associated genes

**Figure 6:**
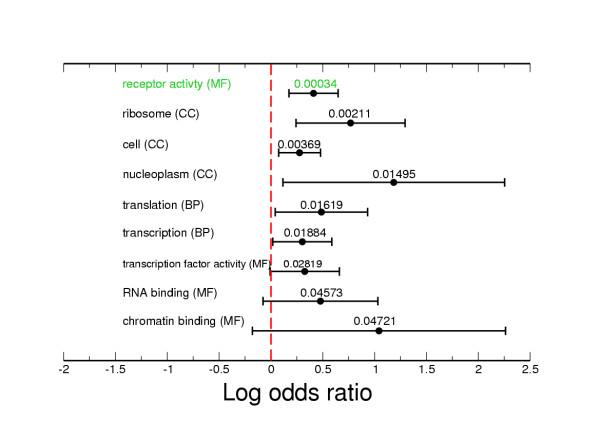
**Over-represented GO terms for *C. elegans *genes with an MRS within 200 bp of CDS stop codon**. The log odds ratios and 95% confidence intervals (two-tailed test) for the top most over-represented GO slim terms for genes with an MRS within 200 bp of the CDS stop codon. The GO terms are split into three ontologies cellular component (CC), biological process (BP) and molecular function (MF). The number above the bar represents the *p *value. Only the term "receptor activity" was significant after correction for multiple testing.

If the MRS is related to a *cis *regulatory function then the presence of an MRS near a gene may be associated with a particular functional group of genes. This possibility was examined by identifying the set of *C. elegans *genes with an MRS within 200 bp of the CDS stop codon, and searching for over-represented Gene Ontology (GO) terms within this set. The top most over-represented GO slim terms are shown in Figure 6. The most over-represented term was the molecular function "receptor activity": 89/509 genes in the MRS set had this annotation (17.5%) compared to 1122/9102 genes in the reference set (12.3%). None of the other terms were significantly over-represented after correction for multiple testing. Analyses were conducted to detect correlation of MRS-associated genes with other genomic and functional genomic features, including expression pattern (as determined by Serial Analysis of Gene Expression data) and position in operons, but no significant associations were obtained (data not shown).

## Discussion

In describing and analysing MRS frequency in the genome of *C. elegans*, we have shown these sites to have a specific distribution, particularly in relation to genes. These observations support the validity of the MRS as a real genomic feature, though not necessarily indicative of MAR, and may also provide an insight to specific roles for MRS.

At the chromosomal level, MRS density had features similar to that of protein-coding genes, with more MRS per kilobase in chromosome centres compared to arms. Chromosome X was distinct in having no such pattern in gene density, and MRS on the X also had a flat distribution. The MRS signature is AT rich, and thus some correlation with local AT% of the genome would be expected (see Additional File 4). We investigated whether the distribution of the MRS signature was merely a by-product of the local nucleotide content of the genome, and/or of the local content of di-, tri- and tetra-nucleotides. When genome sequence was randomised in 2 Mb sections, the frequency of MRS observed in the real chromosomal DNA was less than that predicted from simple (mononucleotide) randomisation, and approximately double that found in second and third order Markov model randomisations. Thus, we conclude that the distribution of the MRS signature in the *C. elegans *genome is not simply a product of small- or large-scale base-compositional biases. MRS frequency in some classes of genomic regions was elevated compared to the surrounding sequence. Coincidence of MRS and genes was apparent from their similar chromosomal distributions (as shown in Figure 1). By analysing the overlap of MRS with different functional parts of the genome, we found that MRS had relatively high incidence in the non-coding parts of genes, specifically 5' and 3' UTRs and introns. These results contrasts with experimental identification of a high incidence of MAR in intergenic and intronic regions, rather than UTRs. This suggests that MRS may not be representative of a large portion of MAR.

There were striking peaks of average MRS frequency at the 3' and 5' ends of *C. elegans *CDS, which were distinct from similar peaks in average AT content in the same regions. Interestingly, the average MRS frequency surrounding *C. briggsae *CDS showed no peak at the 5' end, though the pattern of average AT content was very similar to *C. elegans*. However, the peak at the 3' end of CDS was maintained in *C. briggsae *and there was evidence for conservation of MRS in this region.

Although *C. briggsae *orthologs of *C. elegans *genes that had 3' MRS were more likely also to have an MRS than were orthologs of genes that lacked an MRS, it was surprising that the MRS was conserved in only 10% of orthologs. It is possible that the MRS, as currently defined, does not accurately represent the potential functional element. The non-conserved MRS from both *C. elegans *and *C. briggsae *could represent a high 'false positive' rate, giving rise to a background level of MRS that masks the degree of conservation of the underlying functional element. Alternatively, the apparent low level of conservation of MRS could reflect rapid evolution of the MRS. The association of MRS with the start and stop of genes means they are in a position to influence the control of transcription. The over-representation of the GO term "receptor activity" in genes with an MRS near the 3' end was significant but small. However, if, as discussed above, the MRS does not accurately represent an underlying functional element and is subject to a high false positive rate, then the true degree of association with specific annotations may be underestimated. Efforts were made to correlate MRS-associated genes with other genomic and functional genomic features, including expression pattern (as determined by Serial Analysis of Gene Expression data) and position in operons, but no significant associations were obtained. The presence of MRS in *C. elegans *5' and 3' UTRs suggest that they may be transcribed and therefore also have a role in mRNA stability or translational control. The MRS is therefore an element that is perhaps of limited value in predicting MAR, but serves as a clear marker of some CDS boundaries.

## Conclusion

We have carried out a genome-wide analysis of the distribution of MRS in *C. elegans*. Two distinct patterns of MRS frequency were identified. MRS were less frequent that would be predicted by nucleotide content but more frequent than predicted by di-, tri and tetra-nucleotide pattern. In comparison to the rest of the genome, there were striking peaks of average MRS frequency flanking *C. elegans *CDS. Although *C. briggsae *surprisingly lacked a peak in average MRS frequency upstream of CDS, *C. briggsae *orthologs showed conservation of MRS in the region immediately downstream of the CDS. The results presented here reveal the MRS to have a non-random genomic distribution, with particularly close association with genes. The results further suggest that, rather than acting as a marker for MAR, the MRS is an indicator of CDS, and may have role in control of gene expression.

## Methods

### MRSfinder

The identification of MRS on a genome-wide scale was automated through the use of a custom perl program, MRSfinder. Using the description of the MRS given by van Drunen *et al*. [5], MRSfinder locates all occurrences of the MRS in a given sequence in either orientation and reports their start and stop positions. The program is freely available [30].

### *C. elegans *genome sequence data

Version WS150 of the *C. elegans *genome was downloaded from the WormBase ftp site [31]. The associated gene annotation for WS150 was downloaded using WormMart [32] and additional annotation was downloaded from the WormBase genome browser [33].

### *C. briggsae *genome sequence data

Version cb25 of the *C. briggsae *genome was downloaded from WormBase ftp site [34]. This version is assembled into 578 contigs. The associated annotation was downloaded using WormMart [32].

### MRS and gene distribution in 2 Mb windows

Each chromosome was divided into consecutive, non-overlapping 2 Mb windows, with the first window starting at chromosome base position 1. Where the final window did not contain 2 Mb, the counts for that window were scaled proportionally. For each window, the number of MRS (from MRSfinder) and gene start positions (from WormBase) were assessed. Where a gene was annotated as having more than one transcript or gene model, one transcript and model was randomly selected.

### Mononucletide randomisation of the genome sequence in variety of window sizes

For randomisation of sequences >= 32,000 bp, a roulette wheel selection algorithm was used where a nucleotide's chance of selection was based on its frequency in the original sequence. Due to the stochastic nature of this randomisation method the nucleotide frequency was verified to ensure it fell within 0.2% of that found in the original sequence. For sequences <32,000 bp, the sequence was randomised using a Fisher-Yates shuffle. Each sequence was randomised 1000 times. Each chromosomal sequence was split into consecutive, non-overlapping windows of the appropriate length with correction for shorter end windows as above. Following randomisation, MRSfinder was used to identify all the MRS in the randomised sequence. The mean and standard deviation of the MRS counts for each randomised version of the sequence were calculated.

### Randomisation of the genome using Markov chain processes

First, second and third order Markov chain processes were used to randomise the genome sequence following the algorithm of Workman and Krogh [35]. In a first order Markov chain process, the first nucleotide is chosen by sampling from the mono-nucleotide frequency. Subsequent nucleotides are added by sampling the probability distribution derived from the frequency of the four di-nucleotides that start with the previous nucleotide. Higher order Markov chain processes are used to generate randomised sequence in a similar fashion.

### Number of MRS in genome features

Genes, introns, exons, 3' UTR and 5' UTR were identified based on the GFF file for the appropriate *C. elegans *chromosome. Intergenic regions were defined as all sections of DNA not annotated as belonging to a gene. Where two or more incidents of a single feature type overlap, they were joined to form a single incident of that feature. The genomic coordinates of each feature were used to identify MRS that lay wholly within and partially overlapping a unit of that feature.

The number of MRS expected to lie wholly within each feature type (i.e. complete overlap) was calculated using the formula:

M(F((f-m)+1))/c

The expected number of MRS expected to partially overlap a feature:

M(F(2(m-w)))/c

When the average size of the MRS exceeds that of the feature, a complete overlap is defined as a feature lying wholly within an MRS. The expected number was calculated using the formula:

M(F(m-f)+1))/c

The expected number of partial overlaps when the average size of the MRS exceeds that of the feature:

M(F(2(f-w)))/c

where M = number of MRS, F = number features of specific type, f = average length of feature, m = average length of MRS, w = minimum number of nucleotides required for a partial overlap and c = chromosome length.

Three different scoring methods were used to combine the number of partial and complete overlaps to give an overall score. In method 1 complete overlaps = 1 point, partial overlaps = 0 points, method 2 complete overlaps = 1 point, partial overlaps = 1 point method 3 complete overlaps = 1 point, partial overlaps = 1/2 point. In all scoring methods, the minimum number of nucleotides required for a partial overlap was 12. An AT content correction factor was calculated based on the ratio of the number of MRS found in random sequence with the same AT content as each feature to the number of MRS found in random sequence with the same AT content as the genome. The number of MRS found in random sequence of specific AT content is shown in Additional File 4.

### MRS frequency across CDS

In this analysis, one CDS per gene was used: where a gene was annotated with multiple transcripts and/or gene models, a single transcript/model was randomly selected to represent the gene. The CDS were then subjected to quality filters to remove poor quality sequence (containing Ns), CDS with insufficient sequence upstream or downstream and CDS that did not start with ATG or end with a stop codon. Of the 20,052 *C. elegans *CDS originally identified, 20,032 passed these filters. The 19528 *C. briggsae *CDS were reduced to 12954 after filtering. Each successfully filtered CDS was then split into consecutive, non-overlapping 50 bp windows, starting 1000 bp upstream of the CDS start site and continuing to 1000 bp downstream of the CDS stop site. The total number of MRS mid-points occurring in each window across all CDS was divided by the number of CDS used to produce a frequency of MRS occurrence in that window.

### AT content across CDS

CDS were selected, filtered and split into consecutive, non-overlapping 50 and 10 bp windows as described above. For each window the AT content was calculated as a percentage of the window length. The mean AT% for each position across all CDS was calculated.

### MRS in *C. briggsae *orthologs

The cb25 version of the *C. briggsae genome *sequence and annotated orthologs to *C. elegans *were downloaded from WormBase. After subjecting the 11,953 orthologs to filtering for length (i.e. sufficient sequence upstream and downstream for further analysis), poor quality (sequence containing Ns), and CDS not starting with ATG or ending in a stop codon, 4132 genes remained. MRSfinder was used to detect MRS within 200 bp of the CDS stop for each of these filtered genes in *C. elegans *and *C. briggsae*. To test for association between a *C. elegans *gene having an MRS and the *C. briggsae *ortholog having an MRS we calculated the log odds ratio (a × d)/(b × c) where a is the number of orthologs with an MRS within 200 bp of the CDS stop codon in *C. elegans *and *C. briggsae*, b is the number of orthologs where an MRS is only found within 200 bp of the CDS stop codon in *C. briggsae*, c is the number of orthologs where an MRS is only found within 200 bp of the CDS stop codon in *C. elegans *and d is the number of orthologs where neither organism has an MRS within 200 bp of the CDS stop codon.

### Functional classification of MRS associated genes

A set of *C. elegans *genes with an MRS within 200 bp of the CDS stop codon was analysed to identify over or under-represented Gene Ontology (GO terms). The Gene Ontology annotation file for *C. elegans *was downloaded from the Gene Ontology website [36]. Following Vavouri *et al*. [37], only GO terms inferred from electronic annotation (evidence code IEA) were used due to the bias of RNAi phenotypes on the GO annotations of *C. elegans *genes. The Perl script map2slim and version 1.2 of the generic GO slim ontology (both available from the GO website [36]) were used to obtain GO slim term association counts for the *C. elegans *gene set. Of the 1057 genes in the set, 509 were associated with a GO slim term. The GO slim term counts for this gene set were compared to a reference set, containing all the remaining *C. elegans *genes. For each GO slim term the log odds ratio (a × d)/(b × c) was calculated, where a is the number of genes in the MRS set associated with the term, b is the number of genes in the reference set associated with the term, c is the number of genes in the MRS set not associated with the term and d is the number of genes in the reference set not associated with the term. To account for multiple testing, the Benjamini and Hochberg method was used to calculate a *p *value threshold for a 5% false discovery rate [38].

## Authors' contributions

AA carried out the analyses and wrote the manuscript. MB assisted with the analyses and writing the manuscript and supervised the project. Both authors read and approved the manuscript.

## Supplementary Material

Additional file 1 Distribution of genes and MRS in *C.elegans *chromosomes at window sizes of 100 kb and 500 kb. Number of gene (black) and MRS (red) start positions in non-overlapping 100 kb and 500 kb windows. To account for short sequence length in the end window, the number of genes and MRS in the last window was scaled. Click here for file

Additional file 2 Correlation between MRS frequency and distance to centre of chromosome. Each 2 Mb chromosome window was given a number based on its distance from the centre of the chromosome. The windows at the far ends chromosome were assigned 1, the next windows towards the chromosome centre were assigned 2 and so on until all windows had been assigned a number. The correlation between the MRS frequency in each window and its number was then calculated using Pearson's r correlation coefficient. Click here for file

Additional file 3 MRS in second order Markov chain randomised chromosome I, II, III, IV, V and X. The chromosomes were randomised in non-overlapping 2 Mb windows using a second order Markov chain process. The average number of MRS over 1000 randomisations (+/- one standard deviation) in the 2 Mb windows (black) is compared with the number of MRS in real sequence (red). Click here for file

Additional file 4 Number of MRS in random sequence of defined AT content. The number of MRS in 2 Mb of random sequence with AT content ranging from 90% to 50% was calculated. Random sequence for each AT value was generated 1000 times, error bars show +/- 1 standard deviation. Click here for file
