# Supplementary figures and images for: Association of the Matrix Attachment Region Recognition Signature with coding regions in Caenorhabditis elegans
Source: BMC Genomics. 2007 Nov 15;8:418. doi: 10.1186/1471-2164-8-418 (PMC2234258; doi:10.1186/1471-2164-8-418)

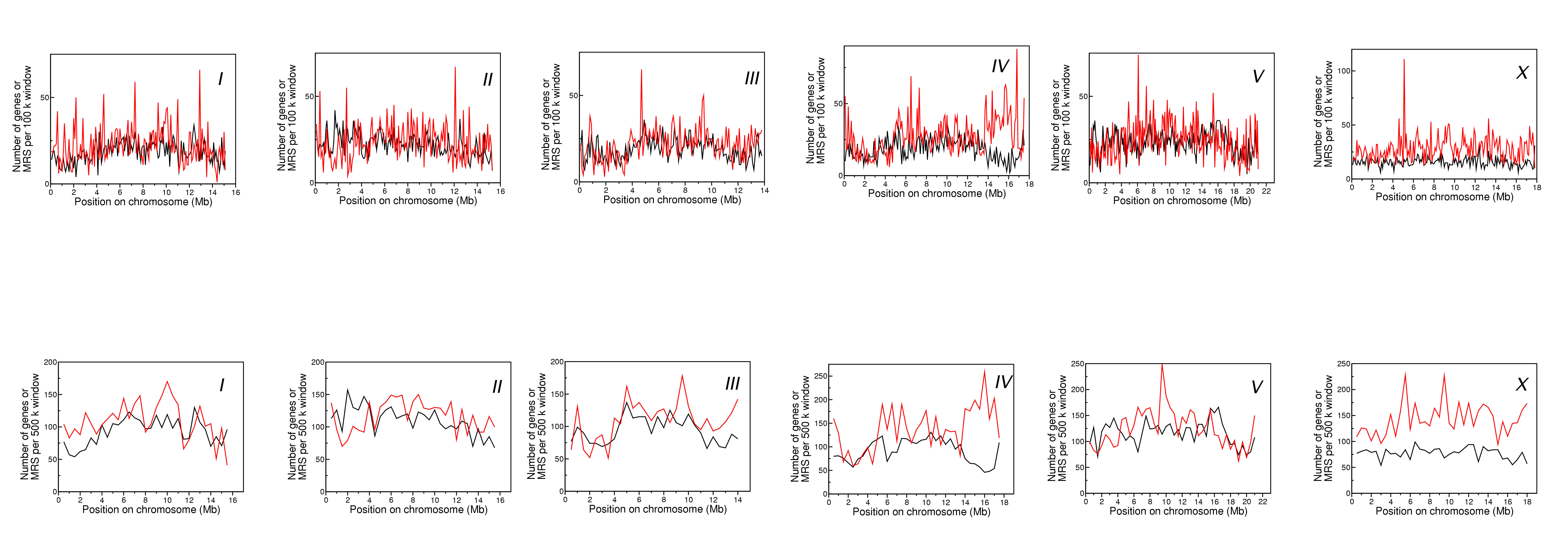

Supplement: Additional file 1 — Distribution of genes and MRS in C.elegans chromosomes at window sizes of 100 kb and 500 kb. Number of gene (black) and MRS (red) start positions in non-overlapping 100 kb and 500 kb windows. To account for short sequence length in the end window, the number of genes and MRS in the last window was scaled. [file 1471-2164-8-418-S1.png]

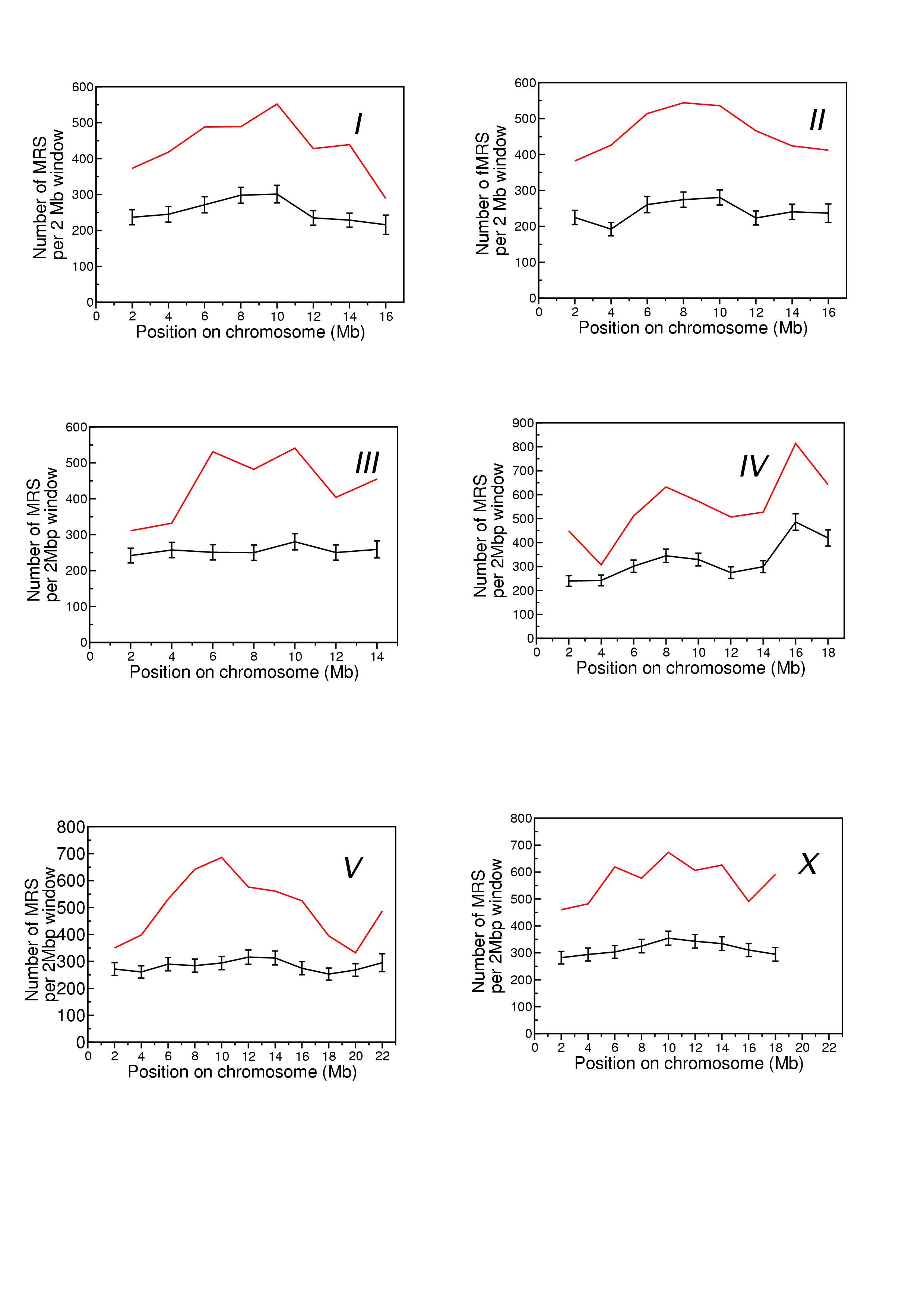

Supplement: Additional file 3 — MRS in second order Markov chain randomised chromosome I, II, III, IV, V and X. The chromosomes were randomised in non-overlapping 2 Mb windows using a second order Markov chain process. The average number of MRS over 1000 randomisations (+/- one standard deviation) in the 2 Mb windows (black) is compared with the number of MRS in real sequence (red). [file 1471-2164-8-418-S3.png]

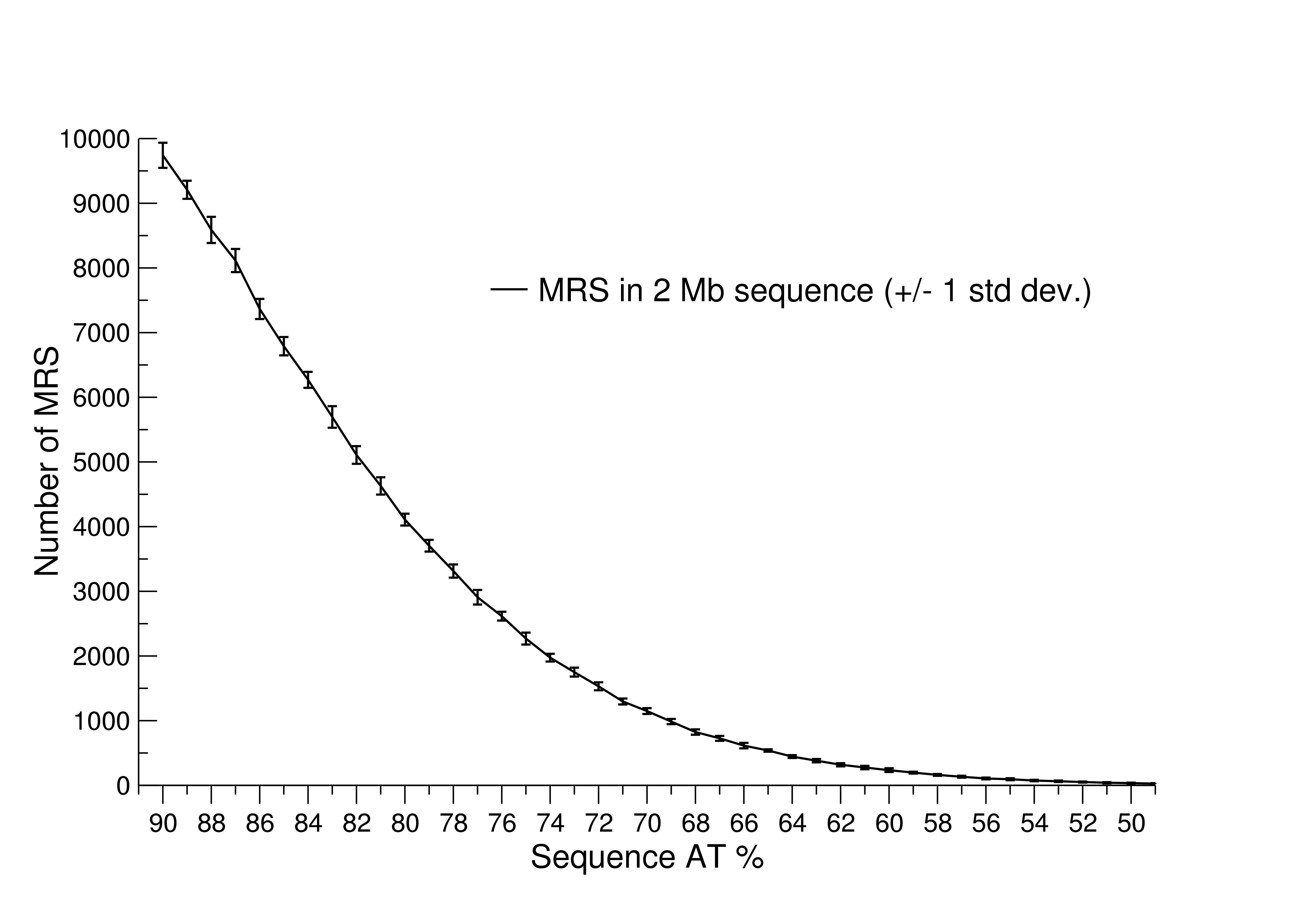

Supplement: Additional file 4 — Number of MRS in random sequence of defined AT content. The number of MRS in 2 Mb of random sequence with AT content ranging from 90% to 50% was calculated. Random sequence for each AT value was generated 1000 times, error bars show +/- 1 standard deviation. [file 1471-2164-8-418-S4.png]
